# Are in‐house diagnostic MR physicists necessary for clinical implementation of MRI guided radiotherapy?

**DOI:** 10.1002/acm2.12171

**Published:** 2017-09-14

**Authors:** Minsong Cao, Kyle R Padgett, Yi Rong

**Affiliations:** ^1^ Department of Radiation Oncology University of California Los Angeles Los Angeles CA USA; ^2^ Departments of Radiation Oncology and Radiology University of Miami School of Medicine Miami FL USA; ^3^ Department of Radiation Oncology University of California Davis Medical Center Sacramento CA USA

## 1 INTRODUCTION

In the most recent years, magnetic resonance imaging (MRI) has showed promising values in MR‐guided radiotherapy (MRgRT) for both brachytherapy and external beam treatments, Thanks to its distinct imaging ability for soft tissue contrast and target identification. However, MRI technology has not been mostly included as part of the required curriculum for a therapy track medical physicist. Lack of sufficient knowledge and proper training may pose obstacles or even safety concerns for those early MRgRT adopters. One of the arguments stands in “the need of hiring in‐house diagnostic MR physicists for clinical implementation of MRgRT.” Herein, we have Dr. Minsong Cao arguing for this proposition and Dr. Kyle Padgett against.

Dr. Minsong Cao is an Associate Professor of Clinical Radiation Oncology at University of California, Los Angeles. He received his Ph.D. in Medical Physics from Purdue University and subsequently worked in the Department of Radiation Oncology at Indiana University before moving to his current position at UCLA. Dr. Cao has been actively involved in the early adoption and clinical implementation of MRgRT at UCLA.

Dr. Kyle Padgett is an Assistant Professor of Radiation Oncology and Radiology at the University of Miami, Miller School of Medicine and is the Director of imaging services for Radiation Oncology. Prior to becoming a clinical physicist, Dr. Padgett was the director of the High‐Field MRI Research Facility at the University of Miami focusing on preclinical research in the areas of cancer and spinal cord injury, among others. He holds a Ph.D. in Medical Physics and an undergraduate degree in Physics from the University of Florida.

## 2 OPENING STATEMENTS

### 2.A Dr. Minsong Cao

With the advent of MRI simulators and MRI‐guided radiotherapy (MRgRT) systems, there have been strong interests in incorporating MRI into radiation therapy (RT) workflow. However, despite its many advantages, integrating MRI into RT workflow is not straightforward due to a number of challenges, which call for strong cross‐disciplinary collaboration between therapeutic and diagnostic medical physicists. Diagnostic physicists with specialized training and clinical expertise in the MRI area play a vital role in the successful clinical implementation of MRgRT. More specifically, their expertise and active contributions are deemed necessary in the following aspects.

#### 2.A.1 Equipment selection and site planning

Although only a few MRI‐guided simulation and treatment systems are commercially available so far, the choice of equipment may be a complex process given the drastic differences in design between systems. To identify a system that is most appropriate requires sound MRI knowledge and a good understanding of the design tradeoffs associated with each system. MRI is a very complex equipment that also requires special site planning and installation considerations including magnetic and radiofrequency (RF) field shielding, data communication channels, and cryogen (e.g., liquid helium) venting in case of an MRI system quench. The MR physicist's knowledge and expertise places him/her in a unique position to liaise with manufacturers, constructors, and users to ensure cost effective purchasing and site planning.

#### 2.A.2 Quality assurance

A vigorous quality assurance (QA) program is essential for maintaining quality and equipment performance of an MRI system. MRI differs from conventional x‐ray based imaging techniques in many ways: its mechanism for producing an image, the types of system imperfections and their impact on the image quality, and the physical and physiological interpretations based on which MRI contrast is generated. Due to these reasons, not all MRI system issues may be identified via routine QA tests. For example, because the geometric distortion of MRI images is not only dependent on the system hardware (e.g., gradient linearity, B_0_ etc.) but also on the magnetic susceptibility and chemical shift effects of each particular patient, a routine phantom QA test would not be able to fully characterize the geometric fidelity of an MRI system. The medical physicist in charge of the QA/QC program needs to have a profound knowledge of MRI technology with an in‐depth understanding of the magnet, gradient and RF coils, MR pulse sequences, and imaging software. Physicist needs to not only master in QA measurements and their results' interpretation, but also understand the limitations of these instruments and procedures. Physicists should also have sufficient clinical experience to identify various issues associated with the MR system, to perform first line troubleshooting and maintenance, and to assist in service engineer to address these issues. An in‐house MR physicist will be a valuable asset to meet the above requirements and to minimize the impact to clinical operation caused by system malfunctions.

#### 2.A.3 MR safety

As pointed out by early MRgRT users,^1^ MR safety is one of the major challenges and concerns in incorporating MRI in radiation oncology, since most staffs are not yet accustomed to a working environment with magnetic fields. Many medical equipment and immobilization devices used in radiotherapy lack clear labels with regard to MR safety and compatibility. Proper MR safety training needs to be provided to all staffs including radiation oncologists, medical physicists, dosimetrists, therapists, and nurses, in order to ensure safety and protection of patients, staff, and general public. Each institution needs to establish an adequate MR safety training program. An in‐house MRI physicist can play an important role in ensuring safety by providing safety training and consultation, developing safety policy and procedures, and overseeing day to day safe operation.

#### 2.A.4 Staff training and education

One of the strengths of MRI is its versatility in offering different types of tissue contrasts using various pulse sequences and image reconstruction algorithms. In addition, the image characteristics and common image artifacts associated with these pulse sequences are also highly variable. To achieve the full potential of MRI, knowledge and experiences in principles of MRI, different pulse sequences and image reconstruction algorithms, as well common types of image artifacts are highly desirable, which are usually not covered in the formal training of radiation therapists and therapeutic medical physicists. An in‐house MR physicist can provide necessary education and training to the RT staff.

Lastly and most importantly, the integration of MRI with RT requires development of special MRI applications with different focus from the diagnostic applications for which image quality is the most important metric. For MRgRT, additional imaging requirements and metrics such as geometric integrity need to be maintained to a higher standard than diagnostic MRI. In addition, imaging with immobilization devices can lead to degradation of image quality due to the increased distance from imaging coil to patient. Therefore, MR pulse sequences and imaging protocols used for diagnostic and staging purpose may not be directly applied to radiotherapy application. Although manufacturers should take primary responsibility of the development of radiotherapy specific sequences and protocols, collaborative or user driven research and development are important paths to facilitate the expansion of MRI into radiotherapy. A MR physicist with expertise in optimizing image sequence can play a vital role in this process. Meanwhile, it is equally important for the MR physicist to have a good understanding of current clinical workflow, practice, and RT specific needs, such as patient positioning and motion management. Mastering the above knowledge requires a strong and persistent commitment, which can be easily achieved by an in‐house MR physicist.

In summary, integration of MRI with radiation therapy is still challenging and requires collaborative efforts and strong cross‐training between radiation oncology and MR physicists. An in‐house MR physicist with specialized training and clinical expertise can play a vital role in facilitating the integration and full adoption of this new technology.

### 2.B Dr. Kyle R Padgett

MRI's role in the diagnosis and treatment of cancer has become prominent in recent years. While originally employed in the diagnosing and staging of cancer, it is now widely utilized in many aspects of cancer care. The soft tissue visualization that diagnostic MRI provides is widely exploited in radiation oncology to greatly assist in the delineation of target structures and in identifying organs at risk more readily than other imaging modalities. It is also used in targeting and guiding of tissue biopsies. MRI is now being integrated into the treatment delivery process with the introduction of MRI equipped Linacs where there are many exciting possibilities. This includes the ability to use superior image quality of MRI for patient setup which increases confidence in aligning the target for radiation treatment. Additionally, these MRgRT devices utilize real‐time MRI during treatment which has many advantages over current gating technologies, and high‐quality MRI datasets facilitate on‐table adaptive RT by providing fast high‐quality three‐dimensional datasets for adaptive planning. These MRgRT devices, developed by ViewRay and Elekta, have been deployed in several clinics already and so this discussion is appropriately timed. I will be arguing that an in‐house MRI physicist is not necessary for the clinical implementation of an MRgRT device.

The implementation of MRI for diagnostic radiology is multifaceted and requires a wide selection of MRI acquisition pulse programs relying on sophisticated and customizable hardware/software which are tailored to the specific needs of the institution: neuro, cardiac, functional MRI (fMRI), and etc. Diagnostic MRI physicists are required to ensure that the correct hardware/software have been purchased and are optimized for the intended needs. MRI physics support is also required for the customization of acquisition protocols to the Radiologist's specifications and for the creation and continual support of a formal QA program which ensures optimal image quality and integrity. For optimal diagnostic MRI performance, a MRI physicist is crucial.

The implementation of MRI for the purpose of MRgRT is significantly different from that in radiology. The imaging associated with these systems has a narrower purpose and the imaging hardware/software have been specifically designed to integrate with the RT system. An example of this is in the design of the ViewRay MRI where specific performance criteria were considered to ensure that the device is appropriate for MRgRT: fast image acquisition, minimal distortion, low‐field MRI to minimize electron return effect, minimally attenuating RF coils, and limited selection of pulse sequences. The imaging system is inherently static and customization of the MRI hardware/software or pulse sequences is minimal without a research agreement.

Establishing a MRI QA program for these systems is a physics responsibility and facilitating this includes daily and monthly QA phantoms and analysis software, provided by the vendor. The routine QA program checks the essential aspects of MRI performance and ensures that the system continues to be suitable for MRgRT. Some of the imaging QA tasks include spatial integrity, MR/RT coincidence, signal‐to‐noise ratio (SNR), contrast, resolution, image scaling, uniformity, and image distortion. Due to the narrowly targeted purpose of the system as well as the suite of QA phantoms and analysis software, I believe clinical therapeutic physicists have the ability to learn the necessary knowledge, if they do not already have it, and to implement the QA program. While previous MR imaging experience would accelerate the therapeutic physicist along the QA learning curve, accreditation as an MR imaging physicist is not essential.

That is not to say that imaging specialists do not play a role in maximizing the effectiveness of these novel treatment devices. Integrating advanced MRI features into the clinical workflow is challenging and if done correctly can greatly benefit patients. Some of these advanced features consist of MRI treatment planning by incorporating electron density information from a CT scan, best utilization of daily setup MRIs for treatment guidance, potential treatment response evaluation from daily MRI acquisitions, and adaptive radiotherapy using deformation of planning contours to daily MRI scans.

The specific purpose of the imaging associated with these systems and the lack of customization mitigate the need for an in‐house MRI physicist. Many of the institutions with these systems have research agreements and utilize MRI physics support to develop future applications while only using the system as it is designed does not require an MRI physicist on a routine basis. An analogy from the recent past is the integration of cone beam CT (CBCT) guidance to linear accelerators. When this technology was new, there was significant participation from diagnostic physics, but as adoption became widespread therapeutic physicists rapidly became knowledgeable and responsible for its acceptance, commissioning, implementation, and QA programs. An additional consideration is the manufacturer's desire to make these systems as smooth as possible to integrate into the clinic, thus these systems are designed for ease of use. Yet, requiring an MRI physicist is nevertheless adding resistance for reaching this goal. Therefore, it is my belief that requiring an in‐house MRI physicist for the implementation of MRgRT systems would not be necessary but active participation from imaging specialists to integrate more advanced imaging features into MRgRT will be welcomed.

## 3 REBUTTAL

### 3.A Dr. Minsong Cao

Both Dr. Padgett and I agree that integration of MRI into radiation therapy workflow is challenging with imaging requirements and constraints that are different from diagnostic radiology. These challenges, if not addressed properly, could undermine the advantages of MRgRT. While it is true, as Dr. Padgett pointed out, that the imaging system of an MRgRT equipment has very specific purpose and design, it is important to note that the MRgRT imaging system is far from “inherently static” and lacks of customization. On the contrary, the acquisition techniques for MRgRT can be highly versatile and customized for different tumor sites and different intended tissue contrast mechanisms. The ability to manipulate the soft tissue contrast using various types of MRI pulse sequences and acquisition parameters is a major strength of MRI that is utilized in diagnostic imaging community and should also be taken advantage of in MRgRT applications. In addition to the primary purpose of anatomical localization and patient positioning guidance, MRI has been shown to be valuable for probing physiological parameters and tumor microenvironment such as tissue perfusion, cellularity and pH, all of which may be tremendously useful for tumor response prediction and adaptive therapy. Even for any given type of pulse sequence, the practical implementation on an MRgRT system may be different from diagnostic MRI systems due to differences in performance requirements and physical constraints. For example, geometric integrity is of utmost importance in MRgRT while many diagnostic MRI applications can tolerate certain degree of image distortion. This difference could mandate modifications in pulse sequences that are prone to geometric distortions when they are used for MRgRT. In addition, the magnetic field strength may be different from conventional diagnostic MRI systems, which could also require certain adaptations in the pulse sequence for MRgRT. To facilitate early clinical adoption, MRgRT manufacturers have been focusing on providing robust but simplified MRI solutions. However, in my opinion, continuous evolvement and development of current MRgRT systems to incorporate a wide spectrum of imaging sequences and advanced applications such as functional MRI is necessary and crucial to prove the clinical values of MRgRT in the long term and to advance it as the mainstream technology for radiotherapy. I agree with Dr. Padgett that manufacturers should take primary responsibility in this process and make every possible effort to develop an easy‐to‐use system. However, it is practically difficult to develop an MRI system that can be used by a person without sufficient knowledge and experiences due to the intrinsic technological complexity of MRI. An experienced MR physicist will play a vital role in the continuous evolvement and maturing of the MRgRT technology, and is an important personnel for centers that are committed to the clinical adoption of such a system.

The analogy of integration of CBCT into radiotherapy is a good example of constant implementation of new technology in the radiation oncology field. It is important to note that CBCT is very similar to CT simulator which had been routinely used in radiotherapy long before the advent of CBCT. Therapeutic physicists are usually well trained and very familiar with x‐ray ‐based imaging techniques. As a result, the learning curve for a therapeutic physicist to master in the x‐ray‐based diagnostic knowledge was relatively short and smooth. The integration of MRI into clinical RT workflow is a similar process, except that the involvement of diagnostic physicists will be much greater. Due to the fundamental differences in physics between MRI and CT is substantial, the training and transition of knowledge from diagnostic MR physicists to therapeutic physicists will take much longer time. As pointed out in the opening statement, it requires collaborative efforts and strong cross‐training between MR and radiation oncology physicists to ensure the successful clinical implementation of MRgRT.

### Dr. Kyle R Padgett

Dr. Cao has written an excellent editorial on many of the ways that an in‐house MRI physicist is beneficial in the development and support of an MRgRT program. While these benefits are meaningful, many clinics may choose to forgo an in‐house MRI physicist if the specific needs can be addressed by other means such as collaboration with MRI experts in the Radiology department. Much of my point of view is informed by the therapy‐related specific purpose and lack of flexibility of these systems from an MRI perspective, as is currently the case. If in the future these systems become more like diagnostic MRI scanners with a vibrant selection of pulse sequences, contrast types, and functional imaging protocols, the need for an in‐house MRI physicist may become significant due to the increased complexity and higher probability of image artifact, spatial integrity, and other issues.

Dr. Cao argues that the in‐house MRI physicist would play a crucial role in the equipment selection and, while the future is uncertain. Currently, there are only two vendors of MRgRT systems (with one pending FDA approval), thereby limiting the selection, possibly for some time. Identifying the key differences between only a couple different systems is a more manageable task and drawing on experience from institutions that have these systems as well as existing personnel with MRI experience will help facilitate a decision. My colleague also brought up the important topic on education and training of the staff in MRI safety, yet many institutions already have policies/procedures established by MRI safety expertise and MRI safety training programs created by their radiology departments. In many cases, collaborating with MRI physicists from radiology, if available, and adapting these existing resources rather than developing a program from scratch may be the best approach. Additionally, the training of the RT staff in appropriate settings and operation of the MR imaging system (field of view, resolution, pulse sequence selection, etc.) is a surmountable task on a dedicated RT system. Collaborating with existing institutional MRI experts may be a better approach to complement the vendor provided training on system operation.

Shifting to a topic we mostly agree on, Dr. Cao presents a great case on the benefits provided by an in‐house MRI physicist for the integration of MRI with RT that is outside the scope of what the vendor offers. Whether it is integrating advanced features into the clinical workflow or collaborating with the vendor to incorporate new pulse sequences, an in‐house MRI physicist has the most to offer in this area. On the other hand, if one wishes to use the device as it is designed and does not have a significant interest in generating research with the system, potentially the second or third wave of MRgRT adopters, then the need for an in‐house MRI physicist is diminished.

Therapeutic physicists are cautious by nature, this is a positive trait, and may want an in‐house MRI physicist as part of the MRgRT team to fill specific expertise gaps. Nevertheless, I believe that therapeutic physicists, with support, can overcome many of the MRI specific obstacles associated with these systems. In the end, each institution will have to make its own decision and some will feel it is necessary to obtain an in‐house MRI physicist while others may draw upon their existing MRI experience, the experience of the early adopters, and the training provided by the vendor to address the unique difficulties related to MRgRT without the need for an in‐house MRI physicist.

## ACKNOWLEDGMENT

Dr. Minsong Cao would like to thank Drs. Peng Hu and Yingli Yang for their insightful inputs and valuable feedback on this topic of discussion.

## CONFLICTS OF INTEREST

There are no conflicts of interests for all authors.
